# p53 Expression as a Diagnostic Biomarker in Ulcerative Colitis-Associated Cancer

**DOI:** 10.3390/ijms18061284

**Published:** 2017-06-16

**Authors:** Kazuhiro Kobayashi, Hiroyuki Tomita, Masahito Shimizu, Takuji Tanaka, Natsuko Suzui, Tatsuhiko Miyazaki, Akira Hara

**Affiliations:** 1 Pathology Division, Gifu University Hospital, Gifu 501-1194, Japan; hern@live.jp (K.K.); nsuzui7@gifu-u.ac.jp (N.S.); tats_m@gifu-u.ac.jp (T.M.); ahara@gifu-u.ac.jp (A.H.); 2 Department of Tumor Pathology, Gifu University Graduate School of Medicine, 1-1 Yanagido, Gifu 501-1194, Japan; 3 Department of Gastroenterology/Internal Medicine, Gifu University Graduate School of Medicine, 1-1 Yanagido, Gifu 501-1194, Japan; shimim-gif@umin.ac.jp; 4 Department of Diagnostic Pathology (DDP) & Research Center of Diagnostic Pathology (RC-DiP), Gifu Municipal Hospital, 7-1. Kashima-tyo, Gifu 500-8513, Japan; tmntt08@gmail.com

**Keywords:** ulcerative colitis, p53, dysplasia, colitic cancer

## Abstract

Ulcerative colitis (UC) is defined as an idiopathic inflammatory disorder primarily involving the mucosa and submucosa of the colon. UC-associated colon cancers (also known as colitic cancers) develop through the inflammation–dysplasia sequence, which is a major problem affecting the prognosis of patients with UC. It is therefore very important to detect malignancy from UC at an early stage. As precancerous lesions arising in UC, there are pathological adenomatous changes, basal cell changes, in situ anaplasia, clear cell changes, and pan-cellular change. It is considered that the mutation of the p53 gene plays a crucial role, and the protein expression of p53 in dysplastic crypts may serve as a good biomarker in the early stages of UC-associated colon carcinogenesis. Immunohistochemistry for p53 is a very valuable diagnostic tool in UC-associated colon cancers. However, protein expression of p53 is not always universal, and additional methods may be required to assess p53 status in UC-associated colon cancers.

## 1. Introduction

The WHO Council for International Organization of Medical Sciences defines ulcerative colitis (UC) as an idiopathic inflammatory disorder primarily involving the mucosa and submucosa of the colon, especially the rectum. Its etiology remains unknown, although immunopathological mechanisms and predisposing psychological factors are believed to be involved. It usually results in bloody diarrhea and various degrees of systemic involvement, as well as an increased propensity for malignant degeneration; furthermore, if prolonged, it affects the entire colon. The treatment of UC is dependent on the severity (i.e., mild, moderate, or severe) from clinical findings [1]. The treatment for mild-to-moderate UC is the administration of salazosulufapyridine and other 5-aminosalicylic acid (ASA) formulations. These drugs are useful for the induction of a remission state and the maintenance of UC. For patients with moderate-to-severe UC, oral or intravenous corticosteroids are useful, and if active UC does not respond to 5-ASA treatment, prednisolone is usually started. Half of all patients with colon-type chronic UC undergo surgery within 10 years after onset.

It is known that UC-associated colon cancers (also known as colitic cancers) develop through the inflammation–dysplasia sequence, which is a major problem of UC threatening the patient’s prognosis. The incidence of UC-associated colon cancers was 1.6–3.7%, and that of all colon types was 5.4% [2,3,4]. The development of UC-associated colon cancers has been shown to take on multiple stages of carcinogenesis, and the process is different from the sporadic adenoma carcinoma sequence [5,6,7,8,9].

The mutation of the p53 gene is a critical genetic change, involved in the early stages of UC-associated carcinogenesis of the colorectum. Overexpression of p53 protein in crypts of the colorectum is usually observed in patients with UC when no dysplasia is histopathologically observed, and is used by pathologists to define a state between regenerative changes and intraepithelial neoplasia. It is also used as a biomarker in predicting the risk of evolution toward malignancy. A high frequency of p53 mutations has been reported to be found in patients with chronic UC with severe disease who were not diagnosed with cancer [10,11,12,13].

Herein, we review the histopathological diagnostic criteria and the importance of p53 expression, which may be a diagnostic biomarker of malignant transformation in UC associated-colon carcinogenesis. 

## 2. Histopathological Diagnosis of UC

Based on clinical symptoms and endoscopic findings, the staging of UC is classified into an active phase and a remission phase. There are some histopathological activity classifications, such as Matts classification [14], the Floren classification [15], the Sandborn classification [16], and the Geboes classification [17], which are based on the inflammatory cell infiltration.

### 2.1. Inflammation

**Figure 1:**
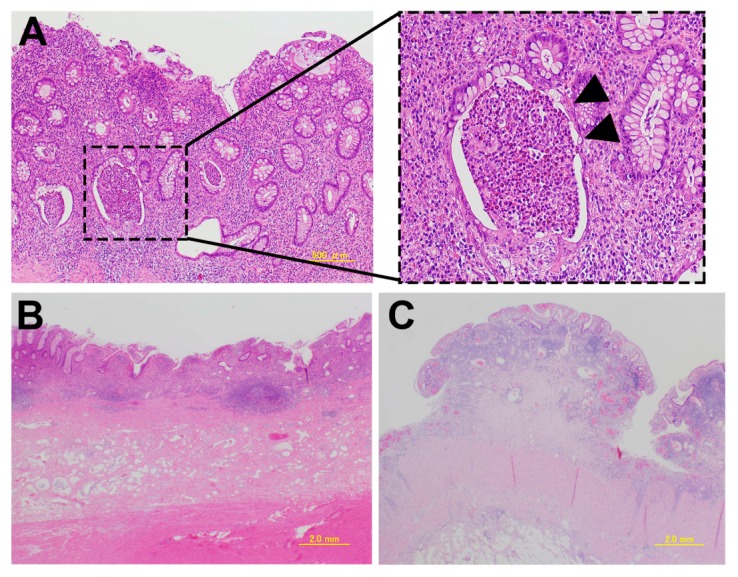
Pathological findings in classical untreated ulcerative colitis. (**A**) A crypt abscess. The arrowhead indicates the crypt abscess; (**B**) Erosion and a decrease in the number of crypts; (**C**) Pseudopolyposis.

Pathological findings in classical untreated UC show the histological pattern of chronic active colitis reflecting active inflammation with the characteristics of chronic mucosal injury. Activity is defined by neutrophil-mediated epithelial injury, with neutrophils infiltrating crypt epithelium (cryptitis), and collections of neutrophils within crypt lumens (crypt abscesses), or by infiltration of surface epithelium with or without mucosal ulceration (Figure 1). The inflammation and mucosal ulceration often cause pseudopolyposis to develop. Chronic changes include architectural distortion, basal lymphoplasmacytosis, and paneth cell metaplasia [18,19]. The architectural distortion includes both shortening and branching of crypts. Basal lymphoplasmacytosis refers to the presence of lymphoplasmacytic infiltration between crypt base and muscle mucosa. Paneth cells are a normal component of the right colon, but their presence in the left colon is a metaplastic change that occurs due to chronic crypt epithelial damage [18,19]. Microscopically, these changes in chronic active colitis are widely homogeneous when symptoms are observed [20].

### 2.2. Dysplasia 

The classification of the Dysplasia Morphology Study Group is widely known as a diagnostic criterion of dysplasia developed in UC (Table 1) [21]. Riddell et al. [22] categorize dysplasia morphologically as adenomatous change, basal cell change, in situ anaplasia, clear cell change, and pan-cellular change. Among these, adenomatous change and basal cell change are most commonly observed [23]. 

Adenomatous change shows some macroscopic appearances, such as a flat and a protruding lesion. It often shows a villous feature and heterozygosity in the bottom of the gland. It tends to differentiate toward the surface layer. There are many unremarkable lesions, sometimes characterized by budding on the surface side and the demonstration of club-shaped villi. Basal cell change is a tissue type common in flat lesions, where relatively small chromatin-rich nuclei are located side by side; this is known as a Beluga caviar-like appearance [22]. Recently, UC has two general patterns of dysplasia, which are commonly classified as adenoma-like dysplasia-associated lesion or mass (DALM) and non-adenoma-like DALM [20,24]. The cytoplasm is broad and eosinophilic. It is poorly differentiated, and has the characteristic that many Goblet cells are unrecognized.

### 2.3. UC-Associated Dysplasia–Carcinoma Sequence

The development of neoplasms in long-standing UC proceeds from nondysplastic mucosa to visible or invisible low-grade dysplasia (LGD), high-grade dysplasia (HGD), and eventually to carcinoma. These UC-associated neoplasms have macroscopically unclear boundaries, and microscopically, dysplasia lesions spread out. Dysplastic lesions invade deeply despite retaining mucosal structures and staying within the mucosal muscularis, compared with normal colon cancer, features that do not significantly affect existing structures. Moreover, there is very little desmoplastic reaction, and there are crypts that infiltrate deeply. Mucinous carcinoma and poorly differentiated adenocarcinoma are considered to be common as the histology of invasive cancer merges with UC [25,26,27].

There is a critical problem with the histopathological diagnosis of UC-associated dysplasia. In the clinical setting, the management of nondysplastic UC (periodic surveillance) or UC-associated HGD (colectomy or endoscopic resection) is currently approved; however, the management of UC-associated LGD is controversial [28,29,30]. It is therefore important to select the most appropriate treatment when dysplasia of any grade is found in a patient with UC. However, it is not often easy to determine the grade of dysplasia.

**Figure 2:**
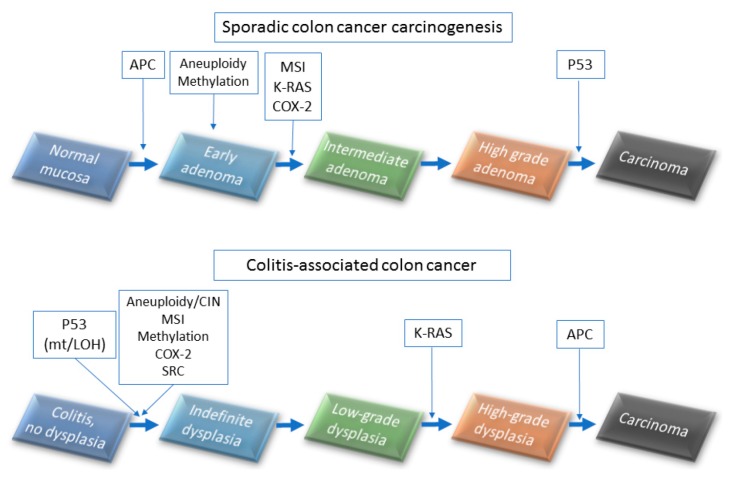
Mechanisms of colorectal cancer and colitis-associated cancer development (upper panel).Sporadic colon cancer carcinogenesis and (lower panel) colitis-associated colon carcinogenesis.

UC-associated colorectal cancers result from a field change effect with multifocal genetic alterations that do not follow the typical adenoma–carcinoma sequence of events (Figure 2). In the typical adenoma–carcinoma sequence, colorectal cancers (CRCs) develop through the accumulation of mutations in several signaling pathways, including WNT, RAS, p53, DCC, and transforming growth factor-β (*TGF*-β) genes [31,32,33]. Adenomatous polyposis coli (APC) mutations are rare events in the UC-associated dysplasia–carcinoma sequence (27.5% of HGD cases) compared with 50% in the typical adenoma–carcinoma sequence [5,34,35]. Tumor necrosis factor alpha (TNFα) is known to be a positive regulator of UC-associated colon cancer, and it is overexpressed in a murine model of carcinoma arising on colitis [36]. Blockade of IL-6, IL-21, and CCL2 have been reported to reduce inflammation-related carcinogenesis in mice [37,38,39]. Negative regulators of UC-associated colon cancer have been reported to be IL-10 [40,41] and TGF-β [39]. Many genes, such as *Bcl-xl*, *kRAS*, *COX*, *iNOS*, *APC*, *Smad3*, *STAT3*, *Ptgs2*, *Tnfrsf6*, *p16*, *Mlh1*, *Runx3*, *Dapk*, and *β-catenin* are mutated in stages of carcinogenesis of the UC-associated cancer [42,43,44,45,46,47].

In the UC-associated dysplasia–carcinoma sequence, *p53* gene mutations are early events in 50% of patients with UC compared with approximately 10% of adenomas related to the typical adenoma–carcinoma sequence [34,48]. A recent study has also reported that p53 immunostaining showed nuclear staining in the basal part of the crypts, even in the indefinite for dysplasia lesions [49]. Wild-type p53 protein in normal cells has a very short half-life [50], and there is no such amount as to be positive by immunostaining. However, abnormal p53 due to the mutation of *p53* is not washed out and accumulates in the nucleus [51]. Therefore, p53—which can be identified as a immunostaining—is basically a mutant p53 protein; conversely, it can estimate gene abnormality of *p53* with overexpression of p53 protein. 

### 2.4. Genetic Alterations of p53 

The *p53* gene encoding the p53 protein and is considered a “genomic guardian” [52]. Loss of defined mutations and heterozygosity (LOH) is observed early in inflammatory carcinogenesis. The LOH means that only one mutation may lead to a complete loss of gene function. This is the case where one allele remains due to previous mutation or inheritance. In addition, a deficiency in patient p53 was observed without signs of dysplasia or neoplasia in more than 50% of colonic tissue specimens of ulcerative colitis, and 50–85% of colitis-associated cancers had defects in *p53* gene [48]. This has been investigated by Brentnall et al. [9], who carefully mapped all resected resection specimens of patients with ulcerative colitis. They have also reported that the mutation of the *p53* gene causes aneuploidy, followed by LOH. That is, p53 detected in the immunostaining is considered the mutation *p53.* p53 immunostaining is widely used as a surrogate for *p53* mutation, however its accuracy has not been reported on colorectal cancer and UC. In ovarian cancers. Optimized p53 immunostaining can approach 100% specificity for the presence of *p53* mutation, and its high negative predictive value is clinically useful, as it can exclude the possibility of a low-grade serous ovarian tumor [53]. Although p53 detected in immunostaining is considered the mutation p53, it has not been established in CRCs related to UC. 

Methods detected for the detection of *p53* mutations are based on genomic DNA or mRNA [54,55,56]. The most widely-used methods are based on a DNA sequencing method. However, several studies compare sequencing assays by using both mRNA and DNA targets [16,57,58,59,60,61]. In the report of whole-exome sequencing analysis of inflammatory bowel disease (IBD)-associated CRCs [36], the mutation spectrum of *p53* was predominantly located in the protein’s DNA binding domain. The spectrum of *p53* single substitution in IBD-CRCs and sporadic CRCs had several noticeable differences. No mutations were observed at hot spot R273, and only one mutation was found in hot spots R248, G245, and R175 in IBD-CRCs but not sporadic CRCs. In IBD-CRCs, the predominant substitution was C:G > T:A transition at CpG dinucleotide (52.6%). This type of substitution at TP53 has previously shown a positive correlation with the expression of enzyme-induced NO synthase (iNOS) in colon tumors, and a reading of inflammation-related DNA damage has been hypothesized.

Recently, next-generation sequencing (NGS) technologies have played a pivotal role in the understanding of the altered genetic pathways in human malignancies. Compared with traditional sequencing methods, NGS technologies have many advantages. NGS is a high-throughput technology, as it permits massive parallel sequencing consisting of the simultaneous sequencing of multiple targeted genomic regions in multiple templates to detect coincident mutations in the same run [62]. The data of p53 mutations analyzed by the NGS will be reported on CRCs associated with UC. 

## 3. p53 Expression as a Diagnostic Marker in UC-Associated Dysplasia

**Figure 3:**
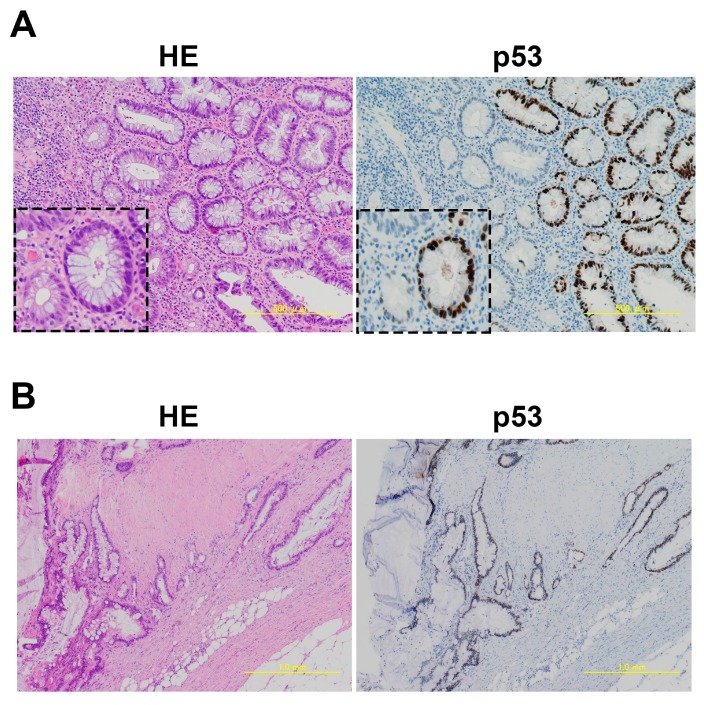
Development of an ulcerative colitis (UC)-associated colorectal cancer from adenomatous change equivalent to high-grade dysplasia. (**A**) Dysplasia adenomatous change associated with UC in HE (Hematoxylin and Eosin) and p53 staining. *Insets* show dysplastic crypts; (**B**) Invasive mucinous adenocarcinoma associated with UC in HE and p53 staining.

Immunostaining of p53 is useful as a tissue biomarker for predicting the risk of renewal changes, differentiation of intraepithelial neoplasms, and the evolution to malignant tumors, including colorectal cancers [49,63,64]. In UC-associated dysplasia, overexpression of p53 protein in the colonic epithelium is also found and detected in cases where the dysplasia is otherwise histologically difficult to determine (Figure 3).

In p53 immunostaining of patients with UC, Noffsinger et al. [65] have reported that there are three patterns which are regularly seen: (1) isolated immunoreactive cells in the crypts base, (2) strongly positive cells confined to the basal half of the glands, and (3) diffusely stained cells [65]. Sato et al. [66] have also reported that the basal pattern of p53 expression is limited to half of the basal cell side, and this pattern is considered pathologically equivalent to LGD or HGD in UC. Kobayashi et al. [67] have focused on the basal pattern of p53 expression and classified the expression on the basal half of the glands into three types: UC-IIa (indefinite for dysplasia, probably regenerative), UC-IIb (indefinite for dysplasia, probably dysplastic), and UC-III (low- or high-grade dysplasia). By visual estimation analyzed with computer-assisted image analysis, p53 basal positivity (more than 20% per the basal half of the crypt) was observed in 46.0% of UC-IIa crypts (128 of 278 cases), 61.9% of UC-IIb crypts (39 of 63 cases), and 94.2% of UC-III crypts (81 of 86 cases) in patients with UC. This result supports that p53 immunostaining might be a useful tool for detecting UC-associated early-stage neoplasia.

In the European Crohn’s and Colitis Organization and the European Society of Pathology, it is recommended to collect at least four or more biopsies as surveillance for every 10 cm of macroscopically abnormal areas [68]. This suggests that we should reduce the chance that too few cells are biopsied, meaning that the level of p53 basal positivity is unable to be calculated. Furthermore, UC-associated cancers usually have a genetic heterogeneity of tumor cells, even in a single tumor mass [46]. Yin J et al. [46] have reported that p53 point mutations were detected in 26 lesions from 20 UC patients with dysplasia and carcinomas, including 18 carcinomas, 6 dysplasia-associated masses, 1 flat dysplasia, and 1 lymph node. 

Immunohistochemistry (IHC) is a quick and easy method for detecting p53 mutations, although there are some discrepancies between the result of IHC and mutation analysis. In a study comparing the immunostaining of p53 with the *TP53* gene mutation, neither the IHC nor the sequencing alone have a full capability to predict p53 status; however, when combined, these two technologies provide a more complete assessment of p53 status in patients with CRC [69]. Although immunostaining of p53 is a very valuable diagnostic tool for detecting the dysplastic change in UC, we must remember that it is not always universal, and additional methods may be needed to correctly assess p53 status in UC-associated dysplasia. 

## 4. Conclusions

Prospective population-based observational cohort studies in patients with UC with an expert pathologist-confirmed dysplasia and carcinoma are needed to better understand the natural history of UC-associated cancers. Currently, the evaluation of p53 status by IHC might be a useful diagnostic biomarker in the diagnosis of UC-associated dysplasia.

## Figures and Tables

**Table 1:** The classification of Dysplasia Morphology Study Group by Riddell et al. [21].

(1) Negative for dyplasia
・Normal mucosa, Inactive colitis, Active colitis
(2) Indefinite for dysplasia
・Probably negative
(3) Positive for dysplasia
・Low-grade dysplasia
・High-grade dysplasia
